# Comparative analysis of fluorescent nanoparticles in beers using size-exclusion HPLC and Brix-normalized fluorescence spectroscopy

**DOI:** 10.3389/fnut.2026.1849696

**Published:** 2026-07-09

**Authors:** Dávid Semsey, Duyen H. H. Nguyen, Gréta Törős, Roland Győri, Áron Béni, József Prokisch

**Affiliations:** 1 Institute of Animal Science, Biotechnology and Nature Conservation, Faculty of Agricultural and Food Sciences and Environmental Management, University of Debrecen, Debrecen, Hungary; 2 Doctoral School of Nutrition and Food Science, University of Debrecen, Debrecen, Hungary; 3 Institute of Life Sciences, Vietnam Academy of Science and Technology, Linh Trung Ward, Thu Duc City, Ho Chi Minh, Vietnam; 4 Institute of Agricultural Chemistry and Soil Science, Faculty of Agricultural and Food Sciences and Environmental Management, University of Debrecen, Debrecen, Hungary

**Keywords:** beer, beverages, carbon dots, fluorescence spectroscopy, Maillard reaction, size-exclusion chromatography

## Abstract

**Introduction:**

Carbon nanodots (CNDs) formed during brewing may contribute to the fluorescence properties of fermented beverages, yet their presence and behavior in commercial beers remain poorly characterized. This study investigated CNDs-related fluorescence in commercial beers using steady-state fluorescence spectroscopy and size-exclusion HPLC with fluorescence detection (HPLC-SEC-FLD).

**Methods:**

Five commercially produced beers - representing dark stouts, abbey ales, dark lagers, and pale lagers - were analyzed for CNDs-related fluorescence. Glycine-derived CNDs were used as a calibration reference to obtain CNDs-equivalent concentrations. Method performance was evaluated for linearity, limit of detection (LOD), and limit of quantification (LOQ). ATR-FTIR spectroscopy was additionally used to characterize the chemical structure of the fluorescent species.

**Results:**

The SEC-FLD method showed good linearity over the range of 6.25–100 mg kg^−1^ Gly-CND equivalents (*R*2 > 0.99), with an LOD of 0.05 mg kg^−1^ and an LOQ of 0.15 mg kg^−1^. Dominant fluorescent fractions eluted at 6.57–6.90 min, corresponding to apparent molar masses of 260,000–330,000 Da, consistent with high-molecular-weight nanocarbon-like assemblies. Dark and semi-dark beers exhibited higher CNDs-related fluorescence than pale lager, while Brix-normalized values revealed that brewing technology and malt roasting intensity, rather than total soluble solids content, are the primary determinants of CNDs-related fluorescence. ATR-FTIR analysis further confirmed the presence of thermally derived, highly oxygenated organic structures consistent with Maillard-derived carbonaceous assemblies.

**Discussion:**

These findings indicate that CNDs-related fluorescent species in beer originate primarily from Maillard-driven thermal processing during brewing rather than from raw material solids content. The combined fluorometric and chromatographic framework developed here provides a reproducible approach for assessing CNDs in fermented beverages, with potential applications in quality control and process monitoring across beverage types.

## 1 Introduction

Carbon nanodots (CNDs) are carbon-based nanomaterials that can form naturally in complex organic matrices, including food and beverage systems. They are typically quasi-spherical particles with diameters between 1 and 10 nm, composed of graphitic or amorphous carbon cores enriched with oxygen- and nitrogen-containing surface functionalities (1, 2). These structural features confer high water solubility, photostability, and excitation-dependent fluorescence, enabling their detection at trace levels in complex matrices (3). In beverages such as coffee, tea, wine, and beer, CNDs are thought to originate from carbon-rich precursors; including carbohydrates, amino acids, and polyphenols; through thermally driven condensation and polymerization reactions associated with roasting, fermentation, or brewing (4–7).

Beer represents a particularly suitable model system for studying food-derived CNDs, as its production involves multiple thermal and redox processes acting on chemically diverse raw materials (8, 9). Maillard reaction products, melanoidins, and polyphenolic compounds generated during malt roasting and wort boiling are recognized contributors to CND formation (10–12). Dark beer styles, produced using more intensely roasted malts, are therefore hypothesized to contain higher CNDs levels and altered fluorescence characteristics compared to pale lagers (13–15). However, systematic comparisons across commercially relevant beer types remain limited, and existing studies often rely on small or regionally constrained sample sets (13, 14).

Fluorescence is a defining property of CNDs and provides a sensitive analytical handle for their detection; however, it also presents methodological challenges. Beer contains numerous intrinsic chromophores—such as polyphenols and caramelization products—that exhibit overlapping UV–Vis and fluorescence spectra, complicating selective CNDs quantification (3, 15). Advanced analytical approaches, including fluorescence spectroscopy combined with size-exclusion chromatography, are therefore required to differentiate nanocarbon-related signals from the bulk organic matrix. The absence of standardized analytical protocols contributes to variability in reported CNDs concentrations across studies (12). HPLC combined with size exclusion chromatography and fluorescent detector (HPLC-SEC-FD) was developed (16).

Beyond analytical considerations, the origin and significance of CNDs in beer are influenced by both processing and post-production factors. In addition to intrinsic formation during brewing, external sources such as packaging materials or storage-induced aggregation may affect measured fluorescence properties (17, 18). From a toxicological perspective, while synthetic CNDs are generally considered to have low acute toxicity, the biological behavior of thermally derived, food-origin CNDs remains insufficiently understood (19, 20). Given the widespread consumption of beer, characterizing the occurrence, concentration range, and physicochemical nature of CNDs is therefore relevant from both consumer safety and product quality perspectives (21).

In this study, the occurrence and optical characteristics of CNDs were investigated in five selected commercial beers representing a range of brewing styles and malt roasting intensities, including dark stouts, abbey ales, dark lagers, and pale lagers (8, 9, 22–24). To the best of our knowledge, this is the first systematic comparative study of fluorescent nanocarbon-like species across five commercially distinct beer styles spanning a range of malt roasting intensities and brewing technologies. While the HPLC-SEC-FLD analytical framework was established in prior work (16), and CND formation has been documented in coffee (7) and pretzel matrices (25), no study to date has applied this approach to compare multiple commercial beer styles simultaneously. In contrast to those single-matrix investigations, the present study: (i) employs Brix normalization as a novel secondary interpretive framework to disentangle CND-associated fluorescence from bulk soluble solids effects; and (ii) demonstrates that brewing technology and thermal processing history, rather than total extract content, are the primary determinants of CND-related fluorescence in fermented beverages.

## 2 Materials and methods

### 2.1 Beer sample selection and storage

**Figure 1:**
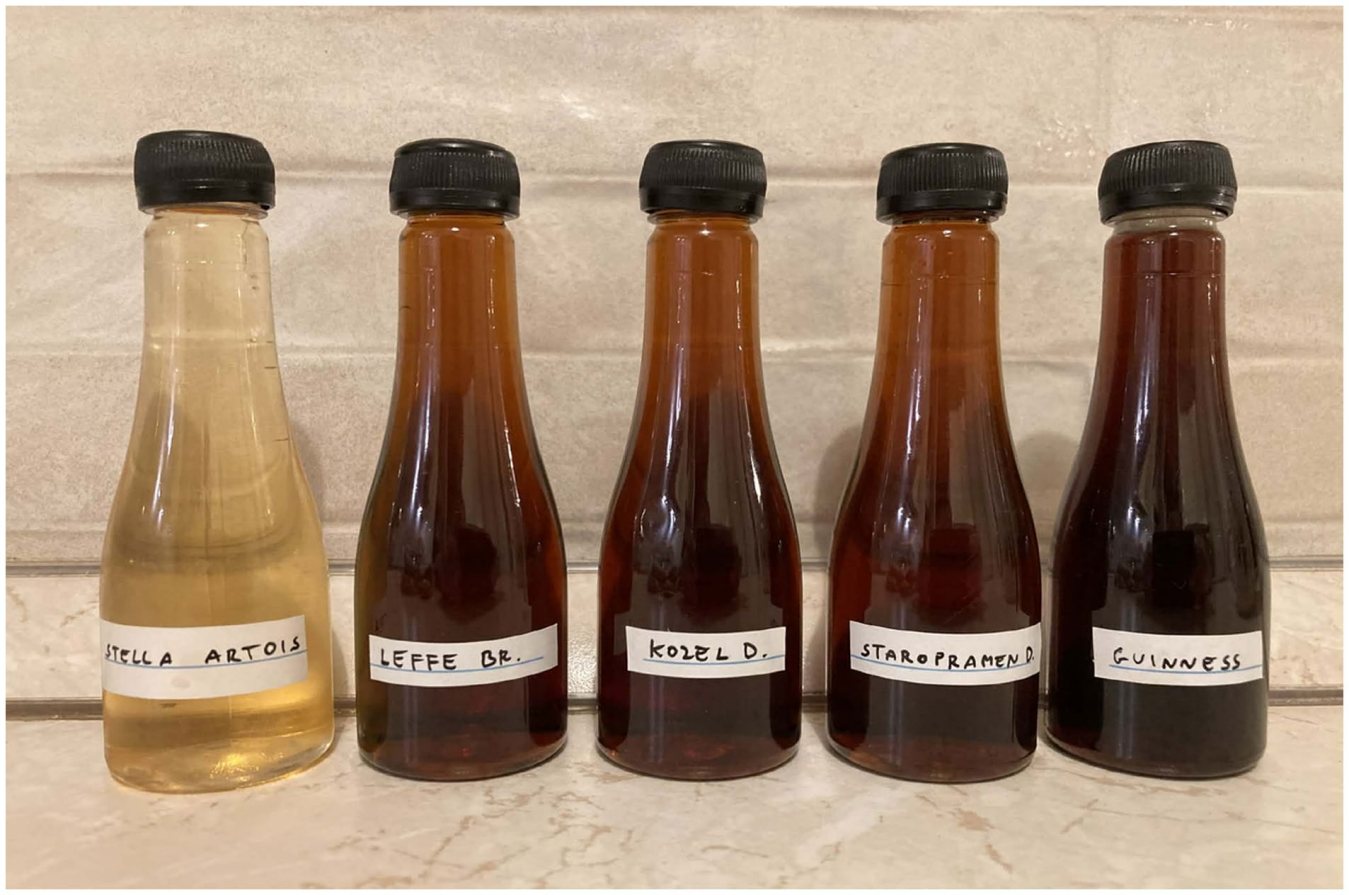
Representative photographs of the five commercial beer samples used in this study, illustrating the visible color differences between dark and pale beer styles. From left to right: Guinness® (stout), Leffe® Brune (abbey ale), Kozel® Dark (dark lager), Staropramen® Dark (dark lager), and Stella Artois® (pale lager).

Five commercially available beers representing distinct stylistic and chromatic categories were selected for this study: Guinness (stout), Leffe Brune (abbey ale), Kozel Dark (dark lager), Staropramen Dark (dark lager), and Stella Artois (pale lager). All samples were purchased from a retail outlet in Hungary (SPAR supermarket, Debrecen). The chosen set was structured to cover a spectrum of malt roasting intensities and brewing technologies: from highly roasted dark malts (Guinness stout; Kozel Dark and Staropramen Dark lagers) through partially roasted abbey ale malt (Leffe Brune) to unroasted pale malt (Stella Artois lager). This range was selected to test the hypothesis that CNDs-related fluorescence correlates with thermal processing intensity rather than beer style alone. No formal statistical exclusion criteria were applied; however, products with known added colorants, artificial flavor extracts, or non-standard adjuncts were excluded to minimize confounding variables. Each beer was stored in its original packaging under dark, temperature-controlled conditions (4–6 °C) until analysis. Samples were analyzed within 1 week of purchase to avoid changes associated with oxidation or storage-related chemical transformations. Bottled products were opened immediately before preparation, and aliquots were withdrawn from the upper portion of the container to minimize potential sediment interference. Contact between the sampling pipette and the bottle cap, label, or neck area was carefully avoided to prevent packaging contamination. A portion of each sample was transferred to transparent plastic containers to document visible color differences (Figure 1).

### 2.2 Reagents and standard preparation

Deionized water (18.2 MΩ·cm, Millipore) was used for all dilutions and chromatographic mobile phases. Analytical-grade reagents, including glucose and glycine (Sigma-Aldrich, Germany), were used to synthesize glycine-based carbon nanodots (Gly-CNDs) as the calibration reference material (13).

Gly-CNDs were synthesized by controlled pyrolysis of a glucose–glycine mixture at 180 °C for 2 h, followed by water extraction, filtration through a 0.22 μm membrane, and lyophilization to obtain a homogeneous, water-soluble powder, according to the procedure described by Nguyen et al. (26). Calibration solutions were freshly prepared before each analytical session by dissolving the dried Gly-CNDs powder in deionized water and serially diluting it over the range of 0.1–10,000 mg kg^−1^.

All glassware and sample containers were acid-washed, rinsed with deionized water, and oven-dried at 80 °C before use to eliminate residual organic contaminants. Amber vials were used throughout to prevent photodegradation of fluorescent components.

### 2.3 CNDs separation by size-exclusion HPLC

Separation of CNDs and related organic fluorophores was performed by size-exclusion chromatography (SEC) under isocratic conditions using a high-performance liquid chromatography (HPLC) system (Ecom Spol. s r.o., Praha, Czech Republic) equipped with a Shimadzu RF-20A fluorescence detector (FLD). Separation was achieved on a TSKgel G3000PWxl column (7.8 × 300 mm, 7 μm; Tosoh Bioscience, Japan) with a matching guard column. The mobile phase consisted of deionized water at a flow rate of 0.7 mL min^−1^, with the column maintained at 30 °C. An injection volume of 1 μL was selected to ensure optimal peak resolution without column overloading. The detector was operated at excitation/emission wavelengths of 350/460 nm. PMT gain was adjusted so that the primary standard signal corresponded to approximately 60% of full scale.

Beer samples were prepared immediately before measurement. Approximately 10 mL of each beer was transferred into a clean borosilicate glass tube and sonicated for 5 min at room temperature (Olympus Endosonic T1711, 2098) to release dissolved CO₂. After degassing, samples were filtered through 0.20 μm syringe filters (Merck, Budapest, Hungary) to remove particulates, yeast cells, or colloidal matter that could interfere with chromatographic separation. Filtrates were transferred to amber HPLC vials, sealed with PTFE-lined caps, and analyzed immediately.

Retention times of 5–8 min were attributed to CNDs with hydrodynamic diameters of approximately 5–8 nm, as determined by column calibration with polyethylene glycol (PEG) standards. Later-eluting peaks (>10 min) were interpreted as smaller low-molecular-weight fluorescent organic species. SEC analysis provided apparent number-average molar mass (Mn) values for the dominant fluorescent fractions. To provide an order-of-magnitude size estimate, Mn values were converted into estimated particle volumes and equivalent spherical diameters using a simplified atomic packing model, with glycine (molar mass 75 g mol^−1^, ~10 atoms per molecule) as the structural reference unit. An interatomic bond distance of 0.14 nm and atomic radius of 0.07 nm were applied, yielding an estimated atomic volume of 0.00144 nm^3^. This model provides only an approximate physical size estimate and does not imply defined molecular structure or chemical uniformity of the fluorescent species.

### 2.4 Steady-state fluorescence spectroscopy

**Figure 2:**
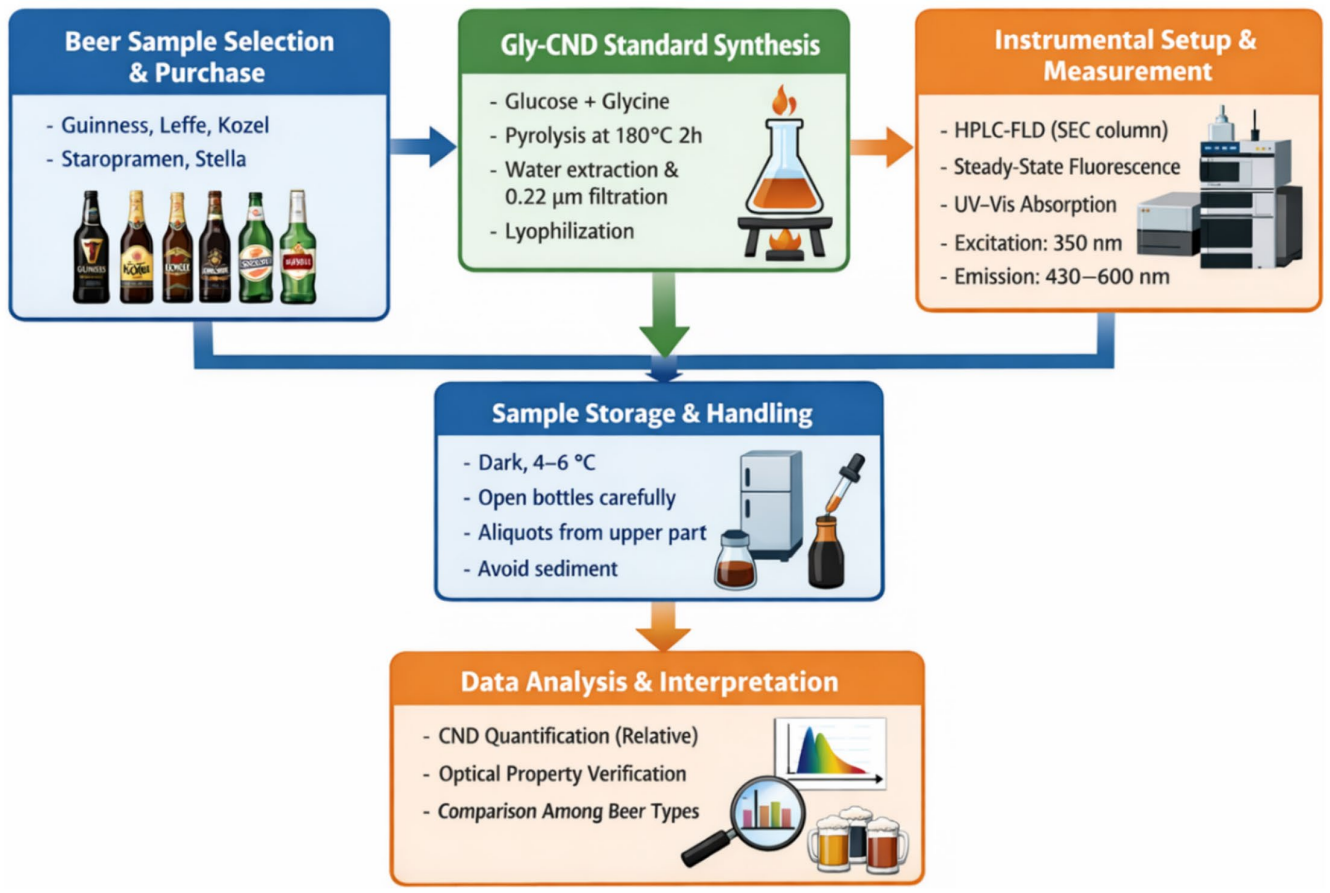
Workflow illustrating beer sample selection, storage, and preparation for CND analysis, including sample handling, SEC-FLD chromatography, and fluorescence spectroscopy steps.

Steady-state fluorescence spectroscopy (SS-F) was employed to evaluate the overall CND-related fluorescence in beer samples. Before measurement, all samples were diluted 180-fold with deionized water to reduce matrix effects and to ensure signals remained within the linear response range. Fluorescence excitation–emission matrices (EEMs) were recorded by scanning excitation wavelengths from 300 to 400 nm in 10 nm increments, while emission spectra were collected over the range 400–650 nm. A primary emission maximum near 407 nm, typically associated with CNDs (26, 27), was targeted; the FLD was operated at Ex/Em = 350/460 nm for comparative analysis. Deionized water was measured as a blank to establish baseline fluorescence. Selected samples were measured in triplicate to assess reproducibility (Figure 2).

**Figure 3:**
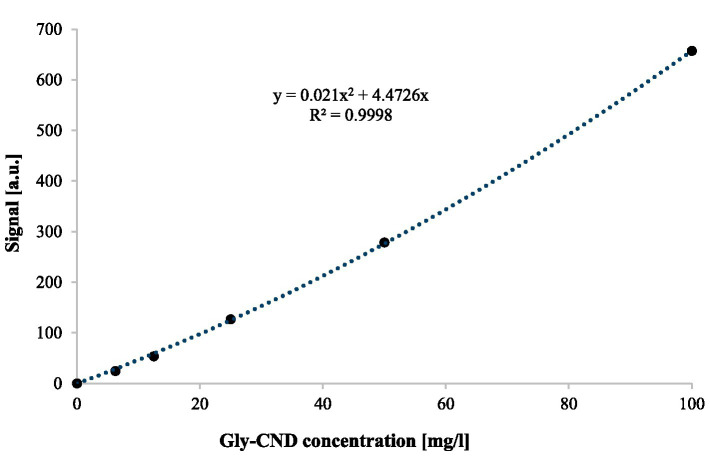
Calibration curve of Gly-CNDs.

A calibration curve was constructed using Gly-CNDs at concentrations of 6.25, 12.5, 25, 50, and 100 mg kg^−1^. The fluorescence response exhibited good linearity within the applied range (*R*^2^ > 0.99; Figure 3). This calibration served as a reference for signal normalization and semi-quantitative evaluation rather than for direct identification of Gly-CNDs in beer. A standard addition approach was applied to selected beer samples by spiking known amounts of Gly-CND into pre-diluted matrices; consistent increases in signal relative to unspiked samples indicated that matrix interference was limited under the applied conditions.

Limits of detection (LOD) and quantification (LOQ) were estimated from repeated blank measurements using LOD = 3*σ*/s and LOQ = 10σ/s, where σ denotes the standard deviation of baseline noise and s is the slope of the calibration curve. UV–Vis absorbance spectra (200–800 nm) were recorded in parallel to assess potential spectral overlap from intrinsic beer constituents such as polyphenols, melanoidins, or other chromophoric compounds (3). Quality assurance included triplicate injections per sample and routine monitoring of retention time drift and signal intensity. Analytical performance parameters are summarized in Table 1.

**Table 1:** Analytical performance summary for the SEC-FLD method.

Parameter	Description/value
Column	TSKgel G3000PWxl (7.8 × 300 mm, 7 μm)
Mobile phase	Deionized water
Flow rate	0.7 mL min^−1^
Column temperature	30 °C
Detection (FLD)	Ex 350 nm/Em 460 nm
Injection volume	1 μL
Calibration range	6.25–100 mg kg^−1^ (Gly-CND equiv.)
LOD/LOQ	0.05/0.15 mg kg^−1^

### 2.5 Determination of soluble solids (Brix)

The soluble solids content of each beer sample was determined using a digital refractometer (TTR95SF, NR151, Dr. Volker Schmidt GmbH, Germany) and expressed as Brix values (°Bx). Before analysis, the instrument was calibrated with deionized water at room temperature. Degassed beer samples were applied directly to the measurement prism, and refractive index values were automatically converted to °Bx. Each sample was measured in three independent replicates, and mean values with standard deviations were calculated. Brix values were used to normalize fluorescence-based CNDs-equivalent concentrations, providing a comparative basis across beers with markedly different extract compositions. This normalization was applied as a secondary interpretative framework to distinguish CNDs-related fluorescence trends from bulk extract effects, not as a correction factor. It should be noted that inner-filter effects were addressed primarily through the 180-fold dilution applied prior to fluorescence measurement (Section 2.4), which reduced sample absorbance at Ex/Em = 350/460 nm to below 0.05 AU in all cases. Brix normalization was applied as a secondary interpretive framework to account for compositional differences between samples, and does not serve as a mathematical correction for concentration-dependent quenching.

### 2.6 ATR-FTIR spectroscopy

Attenuated total reflectance Fourier-transform infrared (ATR-FTIR) spectroscopy was performed on selected beer samples using an Agilent Cary 630 FTIR spectrometer equipped with a single-reflection diamond ATR crystal (Agilent Technologies, Santa Clara, CA, USA). Spectra were recorded over the wavenumber range of 4,000–650 cm^−1^ at a spectral resolution of 4 cm^−1^, with 32 co-added scans per spectrum to improve signal-to-noise ratio. Background spectra were collected against air prior to each measurement. Beer samples were applied directly onto the ATR crystal without prior dilution, and spectra were collected at room temperature. Data acquisition and processing were performed using MicroLab software (Agilent Technologies).

## 3 Results

### 3.1 Separation and identification of CNDs by SEC

#### 3.1.1 Detection window and retention time

A total of 21 chromatographic peaks were detected across all five beer samples. Based on preliminary experiments (25, 26), fluorescent species consistent with carbonaceous nanostructure-like materials were found to elute between 5 and 8 min. Subsequent quantitative evaluation therefore focused exclusively on the major peak present within this retention-time window for each sample. Peaks eluting outside this region were excluded from comparative analysis, as they are associated with smaller, low-molecular-weight fluorophores not relevant to the current investigation. All five beer samples exhibited a dominant fluorescent peak within the targeted window, with retention times showing minimal variation (6.57–6.90 min), confirming consistent elution of the major fluorescent species across samples. Under the applied column conditions (TSKgel G3000PWxl, deionized water, 0.7 mL min^−1^, 30 °C, PEG calibration), the 5–8 min retention window corresponds to apparent molar masses in the range of approximately 260,000–330,000 Da, consistent with high-molecular-weight nanocarbon-like assemblies or melanoidin-associated complexes. Peaks eluting beyond 10 min correspond to apparent masses below approximately 10,000 Da, attributed to low-molecular-weight fluorophores such as small Maillard reaction products or simple polyphenols (16).

#### 3.1.2 Molecular weight distribution

**Figure 4:**
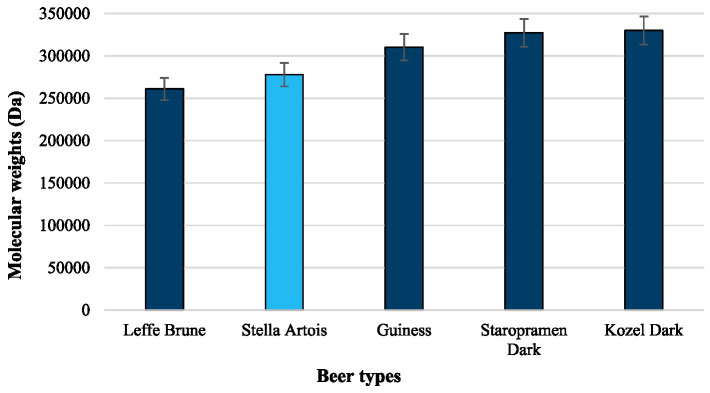
SEC-derived apparent molecular weights of the first eluting chromatographic peaks in the analyzed beer samples.

Substantial differences were observed in both apparent molecular weight (Mn) and fluorescence intensity across samples. Mn values ranged from 260,936 to 329,919 Da, with a mean of 301,623 ± 28,245 Da (SD) and a median of 310,190 Da (Figure 4). This relatively narrow spread, compared to the full chromatographic dataset, indicates that the high-molecular-weight species enriched in this early-eluting window form a distinct and compositionally comparable group across the analyzed beers. It should be emphasized that SEC-derived Mn values reflect hydrodynamic volume rather than absolute molecular mass; the reported values should therefore be interpreted as apparent size-based estimates.

The apparent Mn range of 260,000–330,000 Da observed for the dominant fluorescent fraction substantially exceeds the molecular weights of major beer macromolecules, including lipid transfer protein 1 (LTP1, ~9 kDa), protein Z (~40 kDa), and simple dextrins (<10 kDa), all of which elute at longer retention times (>10 min) under the applied conditions. Furthermore, the fluorescence detector operated at Ex/Em = 350/460 nm provides additional selectivity: beer proteins and typical dextrins are not significant fluorophores at these wavelengths, whereas carbonaceous nanostructures and high-molecular-weight Maillard reaction products are well-characterized emitters in this spectral region. The combination of fluorescence-selective detection and size-based separation therefore provides a two-dimensional discrimination that strongly supports the selective detection of high-molecular-weight nanocarbon-like fluorescent species in the 5–8 min retention window.

#### 3.1.3 Fluorescence intensity

**Figure 5:**
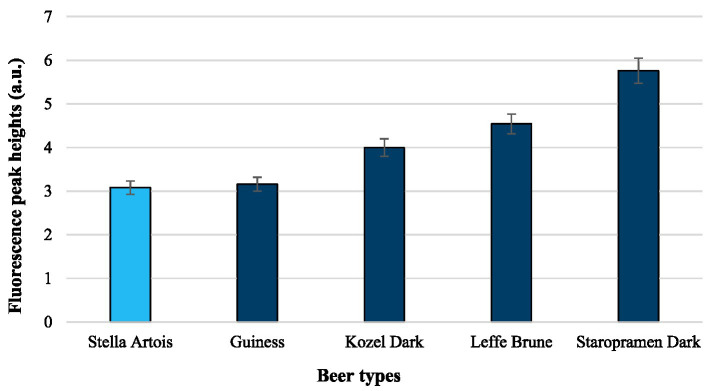
Fluorescence peak heights of the dominant nanocarbon-related fraction (5–8 min) in the analyzed beer samples.

**Figure 6:**
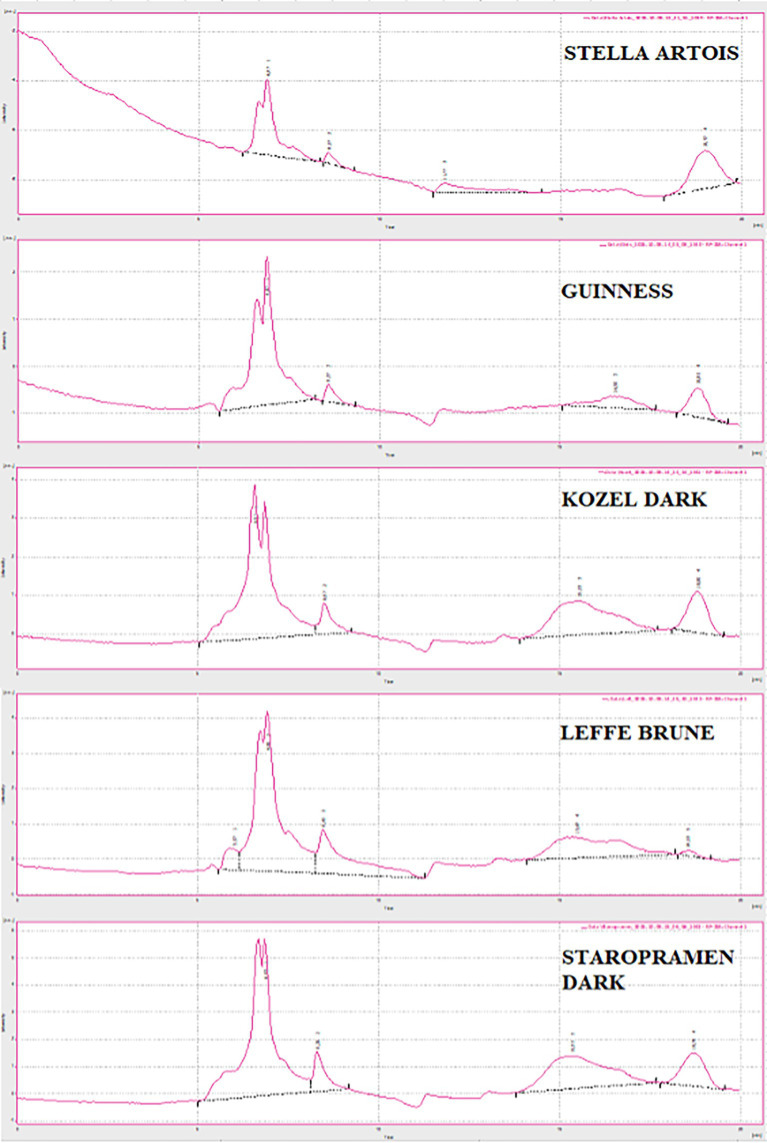
SEC-FLD chromatograms for all five beer samples.

Fluorescence peak heights exhibited greater variability than Mn values, ranging from 3.08 to 5.76 a.u., with a mean of 4.11 ± 0.98 a.u. and a median of 4.00 a.u. (Figure 5). The highest intensity was detected in Staropramen Dark (5.76 a.u.), while Stella Artois showed the lowest value (3.08 a.u.). The monotonic increase from Stella Artois through Staropramen Dark suggests notable sample-dependent differences in the abundance or quantum yield of the fluorescent species eluting in this retention window. The retention-time uniformity, combined with pronounced inter-sample differences in Mn and fluorescence intensity, indicates that the 5–8 min peak cluster provides a robust basis for comparative assessment of nanocarbon-like fluorescent components across beer types. Representative chromatograms are shown in Figure 6.

#### 3.1.4 Size estimation of nanostructures

Using the glycine-based atomic approximation model, Mn values were converted into estimated particle volumes and equivalent spherical diameters. Calculated particle volumes ranged from approximately 50 to 63 nm^3^, it must be emphasized that these diameter estimates are purely theoretical approximations derived from a simplified atomic packing model, and should not be interpreted as direct morphological evidence for nanomaterial identity. Direct size confirmation by TEM, DLS, or AFM remains an essential next step. Among the analyzed samples, Leffe Brune exhibited the smallest estimated particle size (4.57 nm), while Kozel Dark and Staropramen Dark showed the largest values (~4.94 nm). Stella Artois and Guinness displayed intermediate diameters of 4.67 and 4.84 nm, respectively. Despite differences in fluorescence intensity and CND-equivalent concentration, the calculated size range remained narrow across all beer types, suggesting that the dominant fluorescent species share a broadly similar structural scale. These estimated diameters fall within the commonly reported size range for CNDs (1–10 nm), supporting their tentative classification as nanocarbon-like entities. Given that SEC reflects hydrodynamic behavior, the calculated sizes should be interpreted as effective dimensions of solvated or weakly aggregated fluorescent complexes; the presence of polymeric carbon structures, melanoidin-associated nanocarbon assemblies, or loosely bound aggregates cannot be excluded.

### 3.2 Fluorescence quantification and soluble solids analysis

#### 3.2.1 Apparent CNDs concentration (unnormalized)

**Figure 7:**
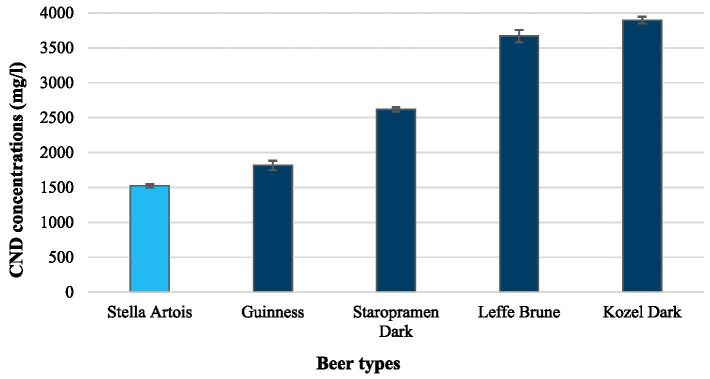
Assumed fluorescence-derived CND-equivalent concentrations in mg/l.

**Figure 8:**
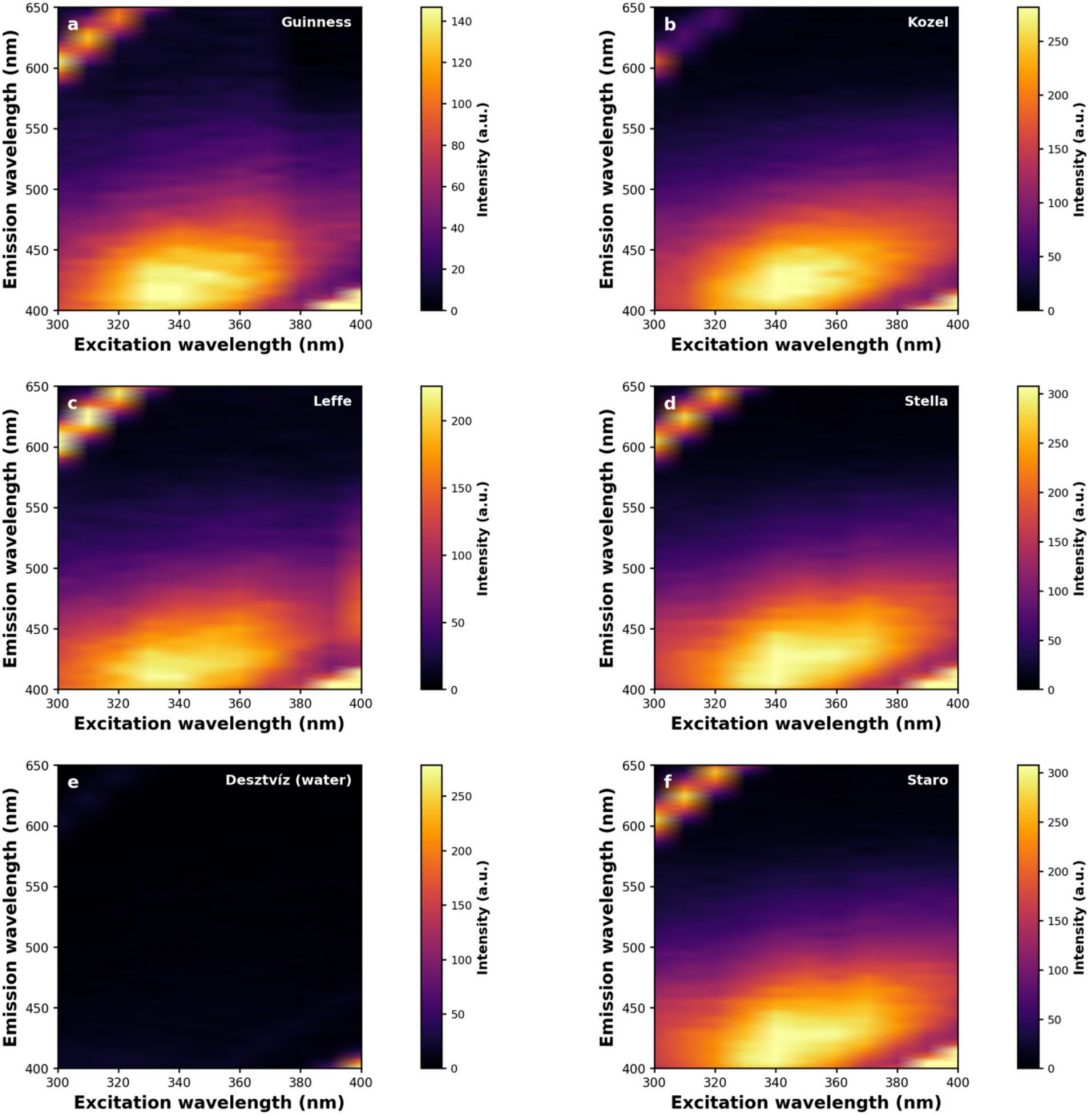
Representative fluorescence emission spectra (*λ* ex = 350 nm) illustrating variations in spectral intensity across different samples: **(a)** Guinness, **(b)** Kozel Dark, **(c)** Leffe Dark, **(d)** Stella Artois, **(e)** water control, and **(f)** Staropramen Dark.

Fluorescence-derived CND-equivalent concentrations were first assessed on a volumetric basis (g L^−1^), independently of soluble solids content. This comparison revealed substantial sample-dependent differences (Figure 7). Dark and semi-dark beers generally exhibited higher apparent CNDs concentrations than the pale lager, consistent with more intensive thermal processing and malt treatment. It should be noted that fluorescence intensity reflects both the abundance and the quantum yield of the emitting species; reported CND-equivalent concentrations therefore represent apparent values rather than absolute nanoparticle counts (Figure 8).

#### 3.2.2 Normalization by soluble solids (Brix values)

**Figure 9:**
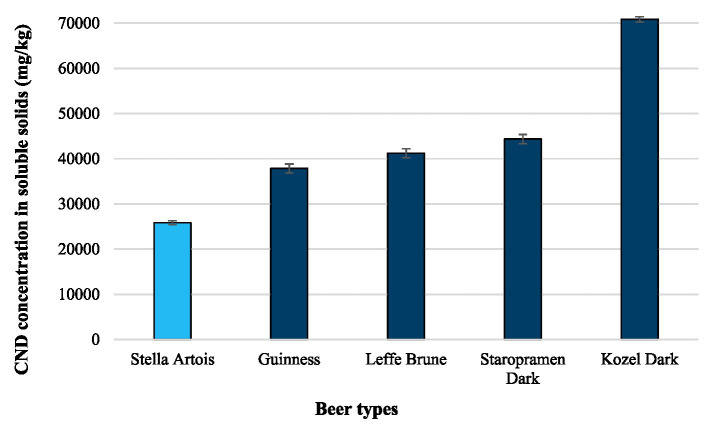
Assumed CNDs concentrations of the different beer samples’ soluble solid contents.

To contextualize concentration values within overall matrix composition, measured fluorescence signals were normalized to the soluble solids content of each beer (Table 2). Brix values ranged from 4.8 °Bx (Guinness) to 8.9 °Bx (Leffe Brune), reflecting substantial variation in soluble solids among samples. While absolute fluorescence intensities ranged from 1,524 a.u. (Stella Artois) to 3,896 a.u. (Kozel Dark), the Brix-normalized values revealed additional trends. Stella Artois displayed the lowest normalized fluorescence (25,830 a.u. °Bx^−1^), whereas Kozel Dark exhibited a markedly elevated value (70,836 a.u. °Bx^−1^), indicating a disproportionately high CND-associated fluorescence relative to its soluble solids content. Intermediate normalized values were observed for Guinness (37,833 a.u. °Bx^−1^), Leffe Brune (41,213 a.u. °Bx^−1^), and Staropramen Dark (39,044 a.u. °Bx^−1^). Notably, despite its highest Brix value, Leffe Brune did not exhibit the highest normalized fluorescence, suggesting that bulk extract content alone does not directly determine CND abundance. These findings indicate that brewing conditions, malt roasting intensity, and thermal history exert a stronger influence on CND formation than total soluble solids concentration. Brix-normalized values are illustrated in Figure 9.

**Table 2:** Brix values, absolute fluorescence intensities, and Brix-normalized fluorescence values for the five beer samples.

Beer	Brix (°Bx)	Absolute fluorescence (a.u.)	Normalized fluorescence (a.u. °Bx^−1^)
Guinness	4.8	1,816	37,833
Kozel Dark	5.5	3,896	70,836
Stella Artois	5.9	1,524	25,830
Staropramen Dark	6.7	2,616	39,044
Leffe Brune	8.9	3,668	41,213

### 3.3 Comparison of HPLC–FLD and fluorescence-based CND quantification

To further contextualize fluorescence-derived CND-equivalent concentrations, SEC–FLD peak heights were compared with steady-state fluorescence-based values. Although both approaches rely on fluorescence detection, they probe fundamentally different aspects of the nanocarbon-containing beer matrix. Steady-state fluorescence integrates the total emissive response of all fluorescent nanocarbon-related species, while SEC–FLD selectively quantifies the fluorescence intensity of a dominant, size-defined fraction within a narrow retention-time window.

Calibration experiments with Gly-CND standards revealed a non-linear relationship between CND concentration and HPLC peak height, best described by a sublinear power-law function H = a·C^b (b < 1), reflecting concentration-dependent quenching, aggregation effects, and detector response limitations commonly observed for CND systems. This calibration indicates that HPLC peak height cannot be interpreted as a strictly linear proxy for absolute CND concentration.

No direct proportionality was observed between fluorescence-derived CND-equivalent concentrations and HPLC peak heights across beer samples. Samples exhibiting high total fluorescence did not necessarily show correspondingly high chromatographic peak intensities, and vice versa. This divergence indicates that the two approaches capture complementary rather than redundant information: fluorescence spectroscopy reflects the cumulative contribution of multiple nanocarbon populations and surface states, whereas SEC–FLD emphasizes the abundance and fluorescence efficiency of a specific high-molecular-weight nanocarbon fraction. The observed discrepancies further suggest that differences among beer samples arise not only from variations in total nanocarbon-like material but also from differences in size distribution, aggregation state, and surface chemistry, all of which influence fluorescence quantum yield. Together, steady-state fluorescence and HPLC–FLD provide complementary perspectives on nanocarbon occurrence in beers.

### 3.4 FTIR spectroscopic characterization

The FTIR spectrum of the beer sample demonstrates the presence of a highly complex organic matrix composed mainly of water, carbohydrates, proteins/peptides, organic acids, alcohols, and thermally generated compounds. The broad absorption band observed in the 3,200–3,500 cm^−1^ region is characteristic of O–H stretching vibrations originating from water, alcohols, phenolic compounds, and hydrogen-bonded hydroxyl groups. This broad band is expected in beer due to its high water content and the presence of ethanol and polyphenolic constituents derived from malt and hops.

The weaker bands around 2,920–2,850 cm^−1^ correspond to aliphatic C–H stretching vibrations, indicating the presence of organic molecules containing methyl and methylene groups, such as carbohydrates, amino acid derivatives, lipids in trace amounts, and Maillard reaction products formed during malting and brewing. The relatively low intensity of these bands is consistent with the aqueous nature of the sample.

In the region between approximately 1,700 and 1,500 cm^−1^, several overlapping absorptions are visible. These signals may be attributed to C=O stretching vibrations of organic acids, esters, aldehydes, and ketones, as well as amide-related vibrations originating from proteins and peptides. Aromatic C=C vibrations from phenolic compounds and melanoidin-like structures may also contribute in this region.

The most dominant feature of the spectrum appears in the 1,200–900 cm^−1^ range, with a strong absorption maximum near 1,030–1,050 cm^−1^, characteristic of C–O, C–O–C, and C–OH stretching vibrations associated with carbohydrates, oligosaccharides, residual sugars, dextrins, and alcohols. The intensity of this band also suggests the presence of thermally modified carbohydrate structures formed during malt roasting and brewing processes. The fingerprint region below 1,200 cm^−1^ contains multiple smaller peaks indicating the presence of structurally diverse compounds including fermentation products, esters, phenolics, and Maillard-derived substances.

**Figure 10:**
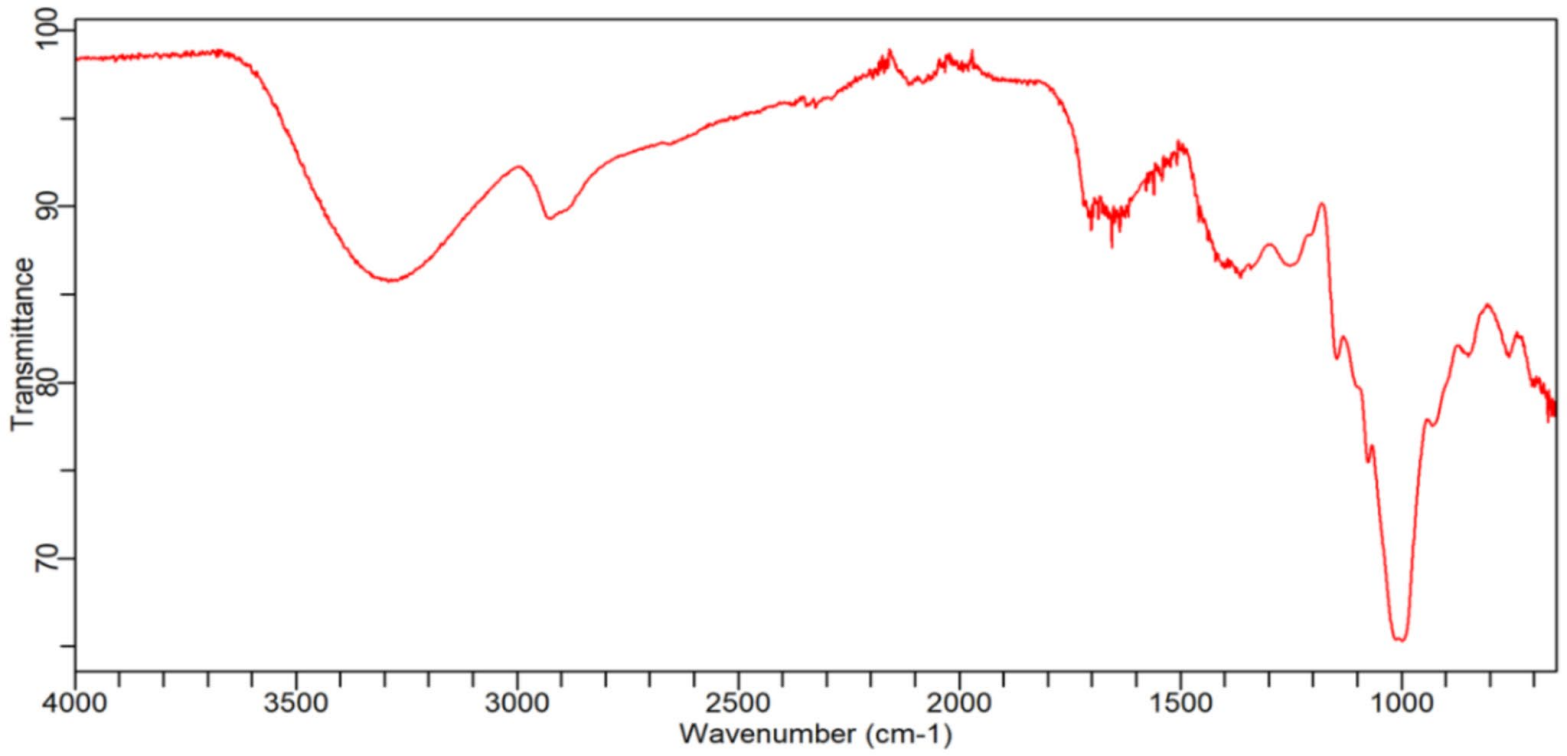
ATR-FTIR spectrum of a representative beer sample (Agilent Cary 630, diamond ATR crystal; 4,000–650 cm^−1^). Key absorption bands are assigned as follows: 3,200–3,500 cm^−1^ (O–H stretching, water and hydroxyl-containing compounds); 2,920–2,850 cm^−1^ (C–H stretching, aliphatic organic compounds); 1,700–1,500 cm^−1^ (C=O and amide vibrations, organic acids and proteins); 1,030–1,050 cm^−1^ (C–O–C stretching, carbohydrates and dextrins). The spectral profile is consistent with a thermally processed, carbohydrate-rich organic matrix compatible with the formation of Maillard-derived fluorescent species.

The FTIR spectrum supports the presence of highly oxygenated organic structures and conjugated functional groups consistent with thermally processed food matrices and compatible with the formation of complex fluorescent carbonaceous structures. The observed spectral features are consistent with Maillard-derived nanoparticles reported in heat-treated food systems (12, 26). However, due to the highly heterogeneous composition of beer, FTIR alone cannot selectively identify CNDs, as many dissolved organic components produce overlapping absorptions. FTIR is therefore considered supportive compositional evidence, complementing the more selective size-based information provided by HPLC-SEC-FLD (Figure 10).

## 4 Discussion

This study provides systematic evidence for the consistent occurrence of fluorescent nanocarbon-related species in industrially produced beers across a range of brewing styles. The combined use of SEC–FLD and steady-state fluorescence spectroscopy enabled both selective chromatographic characterization and bulk fluorimetric quantification, overcoming key limitations of each technique used alone. The SEC retention-time window of 5–8 min, selected based on prior work with food-derived CNDs (25, 26), yielded a dominant fluorescent fraction in all five beers with highly reproducible retention times, supporting methodological consistency. The HPLC-SEC-FD was based on the published work (16).

The apparent Mn values and estimated equivalent diameters are consistent with the size range commonly reported for food-derived carbon nanodots (1–10 nm) (1, 3, 10). The narrow size range across beer types with markedly different compositions supports the interpretation that a broadly comparable class of nanocarbon-related fluorescent species forms during brewing, irrespective of beer style. However, caution is warranted in equating SEC-derived hydrodynamic size with particle diameter, as nanocarbon–melanoidin complexes, polymeric aggregates, or loosely associated nanostructures may co-elute in this window (15). Structural confirmation by complementary techniques such as transmission electron microscopy, X-ray photoelectron spectroscopy, or dynamic light scattering would strengthen this interpretation in future work. In the present study, ATR-FTIR spectroscopy was employed as a complementary characterization tool, confirming the presence of highly oxygenated, thermally derived organic structures consistent with Maillard reaction products and carbonaceous assemblies; however, the technique cannot selectively identify carbon nanodots in a complex beverage matrix.

The observation that dark and semi-dark beers generally exhibited higher fluorescence intensities than the pale lager is consistent with the hypothesis that malt roasting intensity and thermal load during brewing are primary drivers of CNDs formation (10, 28, 29). Notably, Brix-normalized values did not follow the rank order of absolute fluorescence intensities, indicating that total soluble solids content alone does not govern CNDs-related fluorescence abundance. These findings indicate that bulk extract content is not the dominant factor controlling CNDs abundance, and that brewing technology and processing history play a more decisive role. This is consistent with reports from other thermally processed food matrices, where carbonization reactions under specific temperature and precursor conditions govern nanocarbon yield rather than total organic content (4, 7, 12).

The non-linear calibration relationship between Gly-CND concentration and HPLC peak height (power-law, *b* < 1) highlights an important methodological consideration. Concentration-dependent fluorescence quenching and aggregation, well-documented for CNDs (3, 30), limit the direct interchangeability of chromatographic peak height and absolute CNDs concentration. This reinforces the need to treat HPLC-derived values as relative descriptors of a size-defined nanocarbon fraction rather than absolute quantitative metrics. The combined use of both methods therefore provides a more nuanced characterization of CNDs in complex beverage matrices.

It must be acknowledged that Brix is an inherently non-selective normalizer, as it reflects total soluble solids without discriminating between fluorescent and non-fluorescent components (31). Dark beers in particular contain significant quantities of highly polymerized melanoidins that contribute to Brix but do not emit fluorescence at Ex/Em = 350/460 nm and may actively quench the fluorescence signal. This creates a systematic bias when comparing dark and pale beer styles: for dark beers, Brix overestimates the soluble solid fraction directly relevant to CND-related fluorescence, potentially leading to underestimation of normalized fluorescence values. The non-monotonic relationship observed between Brix values and normalized fluorescence across the five samples (e.g., Leffe Brune highest Brix but not highest normalized fluorescence) is consistent with this interpretation (32). Future studies should consider more selective normalization approaches, such as total dissolved organic carbon (TOC) measurement or specific quantification of Maillard reaction intermediates, to provide a more chemically meaningful basis for inter-sample comparison.

From a nutritional and food safety perspective, the widespread occurrence of fluorescent nanocarbon-like species in commercially produced beers raises important questions regarding chronic dietary exposure. Beer is among the most widely consumed fermented beverages globally, and regular consumption may represent a significant and underappreciated route of dietary intake for these thermally derived species. Several studies have reported antioxidant and free-radical scavenging properties for food-derived CNDs and melanoidin-associated carbonaceous species, suggesting potential health-beneficial effects at low exposure levels (12, 18). However, the biological behavior of food-origin CNDs, including gastrointestinal stability, intestinal absorption, systemic bioavailability, and potential interactions with the gut microbiome, remains insufficiently characterized (18–20). Given the structural and chemical diversity of thermally derived nanocarbon species across different beer styles, as demonstrated in the present study, future research should prioritize toxicological profiling, bioavailability assessment, and structure–activity relationship studies to clarify the implications of chronic dietary intake of beer-derived fluorescent nanocarbon-like species.

A limitation of this study is the small sample size (*n* = 5 beers). While the selected samples represent a meaningful range of brewing styles, broader conclusions regarding the full diversity of commercially produced beers should await studies with larger, more geographically diverse sample sets. Future studies should also consider toxicological and bioavailability assessments, given that the biological behavior of thermally derived food-origin CNDs remains insufficiently characterized (18–20).

## 5 Conclusion

This work demonstrates that fluorescent carbon nanodots (CNDs)-related species are intrinsic constituents of industrially produced beers and that their occurrence is strongly modulated by brewing technology rather than by total soluble solids alone. The combined application of HPLC-SEC–FLD and steady-state fluorescence spectroscopy, with Brix normalization as a secondary interpretative framework, provides a robust and reproducible analytical approach for studying naturally formed nanocarbon-like species in complex beverage matrices.

Apparent particle diameters of 4.57–4.94 nm, consistent with known CNDs size ranges, were estimated across all beer types, while fluorescence intensities varied markedly—suggesting that formation efficiency, surface chemistry, and aggregation state, rather than bulk extract content, govern CNDs-related optical signatures. The use of Gly-CNDs as a calibration reference enables comparative CNDs-equivalent quantification without implying structural identity, addressing a key methodological gap in food nanomaterial analytics.

From a technological perspective, malt roasting intensity, thermal load, and overall processing history appear to be the primary determinants of nanocarbon formation in beer, suggesting that CNDs-associated optical signatures could serve as indirect markers of thermal processing intensity or product differentiation. Beyond analytical relevance, the widespread occurrence of nanocarbon species in beers raises important questions regarding dietary exposure and biological interactions. Future studies should integrate toxicological, bioavailability, and structural characterization assessments to clarify the implications of chronic dietary intake of naturally formed CNDs.

## Data Availability

The raw data supporting the conclusions of this article will be made available by the authors, without undue reservation.
